# Intra- and interspecific variation in self-control capacities of parrots in a delay of gratification task

**DOI:** 10.1007/s10071-021-01565-6

**Published:** 2021-10-21

**Authors:** Désirée Brucks, Matthew Petelle, Cecilia Baldoni, Anastasia Krasheninnikova, Eleonora Rovegno, Auguste M. P. von Bayern

**Affiliations:** 1 Max-Planck-Institute for Ornithology, Eberhard-Gwinner-Str., 82319 Seewiesen, Germany; 2 Max-Planck Comparative Cognition Research Station, Loro Parque Fundación, 38400 Av. Loro ParquePuerto de la Cruz, Tenerife Spain; 3 Animal Husbandry, Behaviour and Welfare Group, Institute of Animal Breeding and Genetics, University of Giessen, Leihgesterner Weg 52, 35392 Giessen, Germany; 4 Research Center for Proper Housing: Poultry and Rabbits (ZTHZ), Division of Animal Welfare, VPH Institute, University of Bern, Burgerweg 22, Zollikofen, Switzerland; 5 Max-Planck-Institute for Animal Behaviour, 78315 Radolfzell am Bodensee, Germany

**Keywords:** Delayed gratification, Parrots, Self-control, Comparative cognition

## Abstract

Forgoing immediate satisfaction for higher pay-offs in the future (delayed gratification) could be adaptive in situations that wild animals may encounter. To explain species-differences in self-control, hypotheses based on social complexity, feeding ecology, brain size and metabolic rate have been proposed. To explore these hypotheses in a comparative setting, we tested three macaw species (neotropical parrots)—great green macaws (*N* = 8), blue-throated macaws (*N* = 6), blue-headed macaws (*N* = 6)—and the distantly related African grey parrots (afrotropical parrots; *N* = 8) in a modified rotating tray task, in which subjects are required to inhibit consuming a constantly available low-quality reward in favour of a high-quality reward that becomes available only after an increasing delay (min. 5 s, max. 60 s). All four species successfully waited for a minimum of 8.3 s ± 11.7 s (group level mean ± SD) with African greys reaching a delay of 29.4 ± 15.2 s, and great green macaws—as best performing macaw species—tolerating delays of 20 s ± 8 s. The best performing African grey individual reached a maximum delay of 50 s, whereas, a great green and a blue-throated macaw tolerated a delay of 30 s max. Females tolerated higher maximum delays than males. Engaging in distraction behaviours enhanced waiting performance across species and all birds were able to anticipate the waiting duration. Our results suggest that both feeding and socio-ecological complexity may be a factor in self-control, but further systematically collected comparative data on self-control of different (parrot) species are required to test the evolutionary hypotheses rigorously.

**Supplementary Information:**

The online version contains supplementary material available at 10.1007/s10071-021-01565-6.

## Introduction

Succumbing to one’s desire for immediate satisfaction may spoil better opportunities in the future, therefore self-control is essential for optimising one’s decision-making and for future planning (Santos and Rosati 2015). Self-control is defined as the capacity to forgo immediate less valuable outcomes in favour of a more valuable but delayed outcome (Beran 2015). Accordingly, through tolerating a costly delay without succumbing to the urge of taking the less valuable outcome, the animals are exerting self-control. One way to assess self-control is by using a delay of gratification paradigm, in which individuals are presented with the choice between an immediately available less valuable food reward and a delayed highly valuable reward. Self-control is only one form of behavioural inhibition, however, it is considered to be more cognitively challenging because the subject not only has to suppress impulsive reactions but also must make a decision as to whether a delayed gain is worth waiting for (see Beran 2015 and Miller et al. 2019a for reviews). Other paradigms that assess additional types of behavioural inhibition, such as tests assessing motor inhibition [regulation of impulsive motor actions, e.g., cylinder task, go-no-go paradigm (Miller et al. 2019a)] or cognitive inhibition (ability to control conditioned or learned responses to choose a conflicting, more rewarding or complex course of action, e.g. reversal learning, stroop task), require solely inhibition of prepotent responses, but do not involve a decision component as it is the case in delay of gratification paradigms. In human children, self-control capacity during infancy has been found to be a good predictor of success in later life (Mischel et al. 1989; Tangney et al. 2004) and has also been related to general intelligence (Duckworth and Seligman 2012; Beran and Hopkins 2018). Over the last decades, there has been a growing amount of comparative research on self-control across nonhuman animals in order to learn about the evolutionary roots and drivers of this capacity and better understanding of the underlying cognitive mechanisms (Beran 2018).

In the wild, self-control could be beneficial to nonhuman species in a variety of contexts, like foraging decisions (Stevens and Stephens 2010), social interactions such as mate choice (Sozou and Seymour 2003), or reciprocity in cooperative actions (Stevens and Hauser 2004). Various factors have been proposed to account for species and individual differences in self-control, ranging from physiological aspects, such as energetic states (“Glucose depletion effect”, Mayack and Naug 2015; Miller et al. 2015), metabolic rate and longevity (Stevens and Mühlhoff 2012; Stevens 2014) to brain size (MacLean et al. 2014; Kabadayi et al. 2016). Additionally, species’ differences in self-control have been explained by social complexity (Amici et al. 2008) as well as foraging ecology (Stevens et al. 2005). For example, Amici et al. (2008) found that across several primate species, behavioural inhibition was positively associated with the level of fission–fusion in social groups, while Stevens et al. (2005) found that variation in self-control between gumnivorous common marmosets (*Callithrix jacchus*) and insectivorous cotton-top tamarins (*Saguinus oedipus*) was best explained by differences in foraging ecology.

In addition to species-level differences, individual characteristics including sex (Lucon-Xiccato et al. 2020), physiological measures including body condition (Shaw 2017) or motivation (van Horik et al. 2017) can influence self-control. Furthermore, individuals that are able to perform distractive behaviours, otherwise known as coping behaviours, have also been shown to increase success in delay of gratification in dogs (*Canis lupus familiaris*) (Brucks et al. 2017a), wolves (*Canis lupus* (Range et al. 2020), chimpanzees (*Pan troglodytes*) (Evans and Berans 2007a; b), and one African grey parrot (*Psittacus erithacus*) (Koepke et al. 2015).

Studies on self-control abilities have been conducted using various paradigms. The two most commonly used delay of gratification paradigms are the *accumulation task*, in which food items accumulate as long as the animal refrains from consuming them and the *exchange task,* in which animal are required to exchange a less valuable reward for a delayed highly valuable reward (see Miller et al. 2019a for a review). One alternative method to study delay of gratification was designed to reduce methodological issues with reward visibility (e.g., self-control capacity may be impaired if the delayed option is not visible, Beran and Evans 2006) and delivery (e.g., self-control in an exchange task may be facilitated if the item to exchange is delivered at the beginning of the task, instead of being available for the whole delay period, Pelé et al. 2010a,b). Here, two food items are placed on a round tray connected to a central rotating engine, and become available to the test individual sequentially, at adjustable intervals (Bramlett et al. 2012). Once a food item is removed the rotation stops. Before the start of each test, both food items are first presented to the subject simultaneously. This enables the subject to make an informed decision about which reward to select as they move within reach. The advantage of this method is that it is less cognitively demanding than the classic exchange task in many respects. The task is intuitive given that the two reward options are always visible and that their movement towards the subject can clearly be tracked. This reduces the concern that the subject may not completely grasp the nature of the task and wait for the best option without necessarily gauging the duration of the delay (Beran et al. 2016). Moreover, the animal can directly obtain the reward from the rotating tray without an intermediate step (e.g., a token to exchange, an experimenter to submit the choice), giving the participant more control over the decision, and making the task potentially less confusing (Beran et al. 2016). To date, the rotating tray task has only been applied to capuchin monkeys (genus *Cebus*) (Bramlett et al. 2012; Perdue et al. 2015), preschool children (age 3–5) and New Caledonian crows (*Corvus moneludoides*) (Miller et al. 2019b).

Given this diversity of species tested in various behavioural inhibition paradigms, a comparative approach to study the evolutionary roots of behavioural inhibition is becoming increasingly feasible (e.g. MacLean et al. 2014). Corvids (e.g. members of the crow family) and parrots stand out among avian taxa for their increased brain size and neuron density (Olkowicz et al. 2016) and are good model species for studying convergent evolution of cognitive abilities due to their comparable cognitive abilities compared to primates and their split from mammals 300 million years ago (van Horik et al. 2012). Despite this long phylogenetic distance to primates, these taxa show enhanced cognitive abilities, from problem-solving to decision-making that often rival those of great apes (Emery 2004; Lambert et al. 2018; Auersperg and von Bayern 2019). In contrast to primates (MacLean et al. 2014) and corvids (Kabadayi et al. 2016), parrots (i.e. great green macaws, *Ara ambiguous*; blue-throated macaws, *Ara glaucogularis*; blue-headed macaws, *Primoloius couloni*; African grey parrot, *Psittacus Erithacus*) performed poorly in a comparative study on motor inhibition, where individuals had to suppress a prepotent motor response (Kabadayi et al. 2017). Delayed gratification studies assessing parrots’ self-control capacities, however, suggest that parrots possess self-control capacities that are comparable to those of primates and corvids (Miller et al. 2019a). Goffin cockatoos (*Cacatua goffiniana*) were able to wait for up to 80 s for a reward of higher quality but only 20 s for a reward of higher quantity in an exchange-based delay of gratification paradigm (Auersperg et al. 2013). Using a very similar procedure as Auersperg et al. (2013), keas (*Nestor notabilis*) waited for 160 s for a high-quality reward (Schwing et al. 2017). In a human-speech based delay of gratification task, a single African grey parrot waited up 15 min to get a higher valuable reward, responding to a verbal “wait” command (Koepke et al. 2015), whereas three African greys were not able to wait for a higher quantity of rewards in an accumulation task (Vick et al. 2010). Also, ravens (*Corvus corax*) and crows (*Corvus corone*), from the corvid family, waited for a reward of higher quality for up to 160 s and 640 s, respectively, in exchange-based delay of gratification paradigms (Dufour et al. 2012; Hillemann et al. 2014). Furthermore, all of these bird species were able to anticipate the upcoming delay duration and decided early on in a trial whether it was worth waiting for the delayed option instead of at an arbitrary point during the waiting time (as assessed in the “giving up times”). For comparison, long-tailed macaques (*Macaca fascicularis*) waited for 600 s (Pelé et al. 2010a), chimpanzees (*Pan troglodytes*) tolerated delays up to 480 s (Dufour et al. 2007), Tonkean macaques (*Macaca tonkeana*) and capuchin monkeys waited for 160 s (Pelé et al. 2010b) and rhesus macaques (*Macaca mulatta*) delayed gratification for 120 s (Evans and Beran 2007a). Nonetheless, it needs to be considered that the comparability of those studies is limited as methodological differences can greatly affect the result. Recent studies have pointed out that differential experimental procedures may produce different results in delay of gratification paradigms (see Miller et al. 2019a and Susini et al. 2020 for reviews) and that there is a lack of correlation between different tasks thought to measure the same behavioural construct (Bray et al. 2013; Brucks et al. 2017a; van Horik et al. 2018; Vernouillet et al. 2018).

In the present study, we aimed at broadening the knowledge about self-control abilities of parrots by investigating the individual and species level variation in a delay of gratification paradigm. We use the rotating tray task (Bramlett et al. 2012), which aims to address confounding factors from other delay of gratification tasks. Contrary to the initial procedure, in which the low-quality reward rotates out of reach after several seconds, we modified this task into a delay maintenance task by making the first choice available throughout the delay. Thus, rather than just resisting to take the low-quality reward at the very start of the trial, individuals must continually abstain from taking it until the higher quality reward can be obtained (similar to the exchange task but without the need to hold the reward in the beak; see Miller et al. 2019a for a review). We selected the rotating tray task because of its suitability for comparative research, and because of the lack of comparative data on parrots. It involves little human contact, minimal training and can be applied to various species independent of morphological differences (e.g. using hand, beak or mouth to grab food); thus, reducing sampling biases (Farrar and Ostojić 2020; Webster and Rutz 2020).

We tested and compared four species, three closely related macaw species (great green macaws, blue-throated macaws, blue-headed macaws) belonging to the sub-family of New world parrots (Arinae) and a distantly related species belonging to the old-world parrot subfamily, African greys, which have previously been tested in other self-control tasks (Koepke et al. 2015; Vick et al. 2010), in a modified rotating tray task with rewards differing in terms of quality across different delay durations (minimum 5 s and maximum of 60 s). The four parrot species involved in this study differ in their feeding ecology and social organisation, but also in terms of phylogeny, which makes them suitable to test existing hypotheses regarding species differences in self-control. The great green macaws and blue-throated macaws can be considered specialists relying heavily on just a few plant species. Whereas the blue-throated macaws feed mainly on the mesocarp of the motaçu palm (*Attalea phalerata*) fruits (Hesse and Duffield 2000) and the great green macaws feed predominantly on seeds of the mountain almond tree (*Dipteryx panamensis*) but switch to other plants if it is not available (Berg et al. 2007). In contrast, the blue-headed macaws (Tobias and Brightsmith 2007) and African grey parrots (Amuno et al. 2007) can be considered as feeding generalists with a more granivorous diet consisting of various types of seeds and nuts. The three macaw species are mainly found in small family groups (Duffield and Hesse 1997; Monge Arias et al. 2003; Herrera et al. 2007; Tobias and Brightsmith 2007; Bouzat and Strem 2012), whereas, the African grey parrots are often observed in large flocks with fission–fusion dynamics and “nursery” groups (Chapman et al. 1993; Martin et al. 2014).

We first tested for differences in maximum delay between species, and although we do not formally test social or ecological variables, differences in species maximum delay may provide initial support for either hypothesis. We also tested how different factors may influence success within sessions. We included an individual’s sex and the residual to the bird’s daily body weight (difference between observed mass and predicted individual regression) as a proxy for food motivation, expecting those hungry individuals, i.e. those individuals that have a negative residual, would show reduced self-control. We also included the proportion of time individuals spent exhibiting specific behaviours (i.e., pacing or manipulating objects in the room) during the delay duration. We chose a range of behaviours to include because we have no specific a priori hypotheses about which behaviours may be considered important for subjects. We expected individuals that exhibit higher proportions of these behaviours to have higher success. In a separate analysis, we tested which behaviours, if any, could be interpreted as strategies for displacing attention towards the reward [so-called coping behaviours (e.g. Evans and Beran 2007b)]. Furthermore, we were interested in finding out whether the parrots are able to anticipate the upcoming delay duration or give up waiting at an arbitrary time point during a trial.

## Methods

### Subjects

We tested 28 parrots owned by the Loro Parque Fundación and housed at the Max-Planck Comparative Cognition Research Station inside the Loro Parque zoo, Tenerife, Spain: eight great green macaws (GGM; one male and seven females), six blue-throated macaws (BTM; five males and one female), six blue-headed macaws (BHM; three males and three females) and eight African grey parrots (AGP; two males and six females). Their age ranged from 5 to 13 years. All parrots had participated in various behavioural studies assessing their physical and social cognitive capacities. The parrots were not involved in any previous studies assessing self-control; however, one study tested for motor inhibition (Kabadayi et al. 2017) and another study assessed their economic decisions in a token exchange task (Krasheninnikova et al. 2018).

### Housing conditions

The different parrot species were group-housed in separate aviaries. The great green macaws were divided by age into two social groups. The six younger individuals (5 years) were housed in two interconnected aviaries and the three more senior birds (6–13 years) stayed in a neighbouring but separate aviary. The blue-throated macaws were housed in two interconnected aviaries in a single social group. All five aviaries were 1.8 × 7 × 3.6 m (width × length × height) and they were half outdoors and half under a roof but with ambient outdoor temperature. The blue-headed macaws were housed together in a semi-open indoor aviary (28.61 m^2^) with ambient outdoor temperature and with access to an outside area with natural light. The African grey parrots were housed together in another separate outdoor aviary (21.41 m^2^). To guarantee exposure to sufficient UV light, all aviaries were provided additionally with Arcadia Zoo Bars (Arcadia 54 W Freshwater Pro and Arcadia 54 W D3 Reptile lamp). All aviaries were situated within the same building as the testing chambers. The birds were fed twice a day with a mix of fruits and vegetables in the morning and evening. In the early evening, they were allocated their individual amount of seeds and nuts that was adjusted to the amount of nuts each bird had already obtained during the tests. Birds were mainly fed in small groups or singularly to reduce any potential food monopolisation or aggression. They had unlimited access to water during the day and were not food deprived for the experiment.

### Experimental setup

Training and testing took place in an indoor testing chamber near the aviaries situated within the ‘Animal Embassy’ area of the Loro Parque zoo, where zoo visitors could watch unnoticed by the parrots/experimenter through sound-buffered one-way mirror windows. All the parrots were hand-raised and subsequently transferred to the aviaries and groups they lived in during this study. The parrots were trained to leave their home aviary, enter a transport cage and were habituated to various procedures and materials used for different behavioural studies. Furthermore, the parrots were well accustomed to interacting with various different people. The birds were tested once or twice per day, depending on availability, either in the morning (11 am–1 pm) and/or in the afternoon (2–4 pm).

**Fig. 1:**
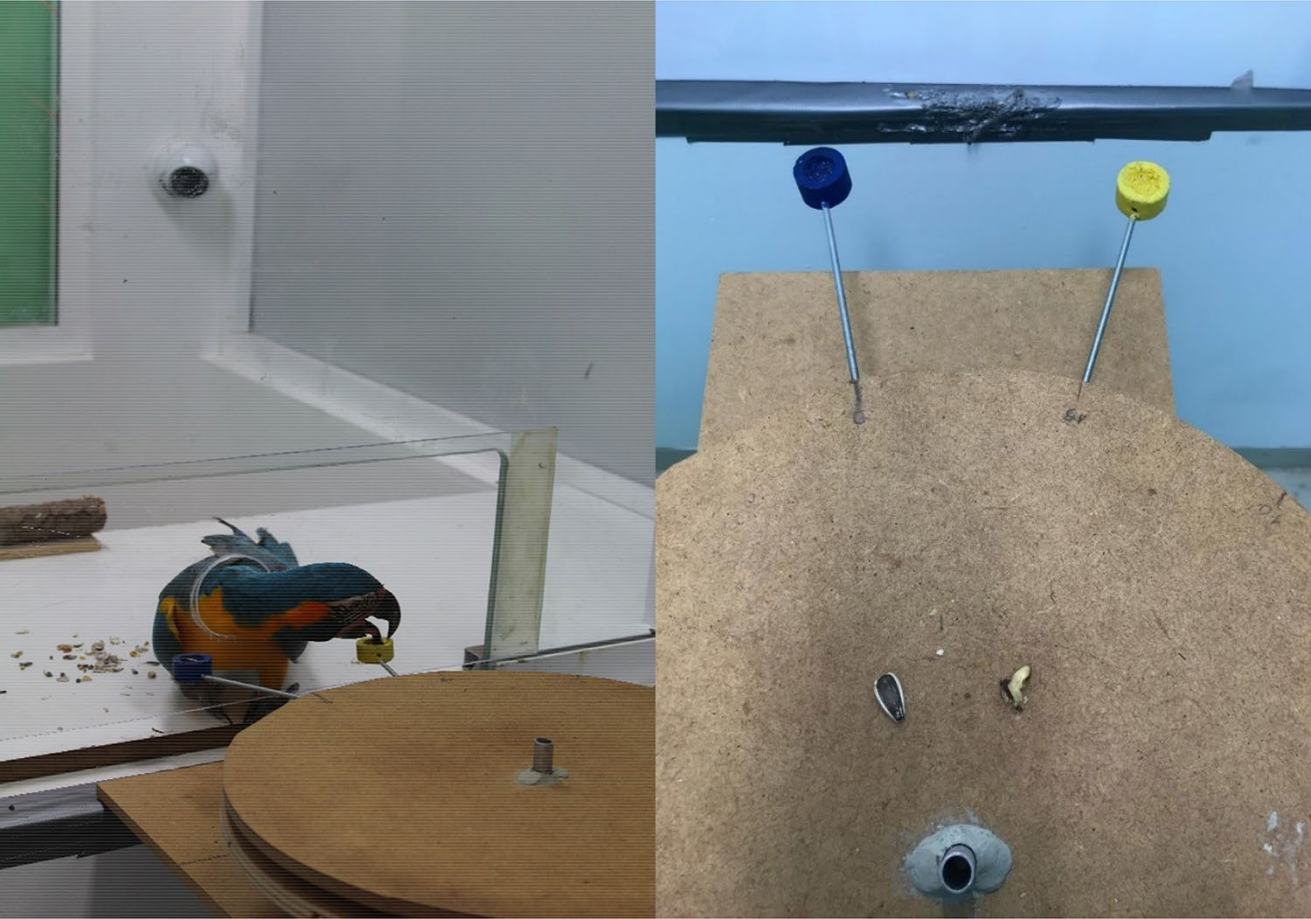
**a** A blue-throated macaw is taking the piece of walnut from the high-quality reward (HQR) food holder after having waited successfully for a certain delay. The sunflower seed remains unconsumed on the blue food holder. **b** Top-view of the rotating disk apparatus mounted on a square wooden tray that can be pulled out of reach. The blue food holder is installed at the low-quality reward (LQR) position, the yellow food holder at the HQR position. The two rotatable chipboard disks (stacked on top of each other) to which the food holders are attached, are shown here in the neutral position as arranged at the beginning of each trial. In the neutral position, the two food holders were equidistant from the circular opening where the subject could retrieve the reward. The two reward types (left: sunflower seed, right: ¼ walnut) are placed on top of the upper disk prior to showing them to the bird

During training and testing, the subjects were placed individually in a testing chamber of 2.5 m × 1.5 m × 1.5 m (height × width × length), while the researcher was seated in the adjacent room with the apparatus. The two chambers were separated by a window (1 m × 1 m) that had a rectangular Plexiglass-covered cut-out (50 cm × 26 cm) at the bottom. A small circular opening (GGM 7 cm diameter; other species 5 cm diameter) cut in the centre of this Plexiglas panel at 5 cm height, allowed the subjects to stick their head into the neighbour chamber and reach the apparatus (see Fig. 1). The testing room contained a table with a wooden perch placed centrally on its edge. One experimenter tested the three macaw species (CB) and another experimenter (ER) conducted the tests on the African grey parrots. The second experimenter (ER) was trained by the first experimenter (CB); however, minimal procedural differences cannot be excluded. Both experimenters were familiar with the parrots and wore sunglasses throughout testing to avoid unintentional cueing of the birds. All training and test sessions were recorded via one camera located in the upper corner of the test room, overlooking the entire table and the window facing the experimenter and apparatus.

### Apparatus

The apparatus consisted of two wooden disks (each of 40 cm diameter*)*, mounted on top of each other. Both disks were attached to a rotating engine (rotation speed of 1 revolution per minute). The whole apparatus was placed on a wooden tray that could be pulled out of reach of the subject (Fig. 1a). A metal rod (15 cm × 0.5 cm) with a round wooden food holder at its end (3 cm × 1.5 cm) was attached to each disk by screwing it into predefined drill holes in the disks. The two food holders differed in colour (blue and yellow) to facilitate reward discrimination. For every single subject, each food holder was assigned to one reward type (high-quality = HQR or low-quality = LQR), keeping it counterbalanced across all subjects as much as possible (e.g. for half the subjects the blue food holder designated high-quality reward and for the other half low-quality reward). Each bird kept this colour allocation throughout the experiment. The food holders could be removed and exchanged, depending on the type of trial (Test or Control) and the subject. Based on a food preference test (see below) we used sunflower seeds as the low-quality reward and walnuts as the high-quality reward.

### General test procedure

At the beginning of every test session, the bird was first weighed on a scale with an attached perch before it was individually placed in the test chamber. The experimenter then moved into the adjacent room, put on sunglasses to avoid cuing the birds and remained passive (i.e. looking straight ahead, no gestures, movements or talking) during testing. An occluder (opaque plastic sheet) covered the window in between trials. To start a trial, the experimenter removed the occluder, gaining the attention of the subject by calling its name before showing both food rewards simultaneously in her hands (between thumb and index finger) in front of the Plexiglass window (at the height of the hole) for 3 s before placing them on the respective food holder. If a bird did not respond to being called by name five times in a row, the experimenter put up the occluder in-between the test rooms and tried again after a break of 30 s. If the bird did not attend to the experimenter following this break, the session was terminated and resumed on the following day. Throughout the test, only one choice (i.e. picking up the reward with the beak) was allowed and as soon as the subject had taken one of the rewards, the other food option was retracted immediately. The window was occluded again after the choice was made to mark the end of the trial.

### Colour preference test

To determine if the parrots had an initial preference for one of the colours, we conducted colour preference tests prior to assigning one colour to the high-quality reward (see Table 1 for an overview).

**Table 1:** Order, summary and criteria of the different test phases

Order	Test phase	Rationale	Conditions	Choice	Trials/sessions	Criterion	Delay
1	Colour preference	Control for colour preference of the food holders	NA	Blue vs. yellow	12 trials	If more than nine choices for either colour = LQR colour; otherwise randomised colour assignment	NA
2	Food preference	Select high-quality food reward	NA	Sunflower seed (LQR) vs. ¼ walnut (HQR)	12 trials per session	At least nine choices for HQR	NA
3	Habituation	Habituation to noise and movement of apparatus	Forced trials	NA	12 trials (six with LQR and six with HQR) per session	Take each reward within 30 s	NA
4	Delay of gratification	Inform on upcoming delay duration	Demonstration trials	NA	Four trials at the beginning of each session	NA	Same as in delay trials
		Assess self-control	Test trials-delay trials	LQR immediately vs. HQR after delay	10 trials per session	Wait for HQR in at least four trials in two consecutive sessions to proceed to next delay	5 s, 10 s, 15 s, 20 s, 25 s, 30 s, 40 s, 50 s, 60 s
		Rule out preference for second food holder	Test trials-LQR control	LQR immediately vs. LQR after delay	Two trials per session	NA	Same as in delay trials
		Understanding of contingencies (= choose HQR)	Test trials-position control	HQR immediately vs. LQR after delay	Two trials per session	NA	Same as in delay trials

During the colour preference test, the experimenter placed the two distinctly coloured food holders (yellow or blue) 20 cm apart from each other on a plastic board. Both food holders were baited with a clearly visible sunflower seed. The plastic board could be pushed through a slot under the window into the testing chamber so that the subject could make its choice.

Twelve trials were performed per individual, pseudo-randomising the position of the coloured food holder, i.e., six trials with the blue food holder on the left and six trials with the blue food holder on the right. To minimise the impact of side biases, one colour could not be on the same side more than twice in a row. Once baited, the food holders were pushed under the window into the experimental chamber.

In case the subject chose equally between blue and yellow, the food holder colour for each reward type was assigned randomly but keeping the colour/reward type allocation counterbalanced across subjects as much as possible. If, however, the subject showed a clear preference for one colour (10 or more choices; *p* < 0.005 in two-sided binomial test), the preferred colour was associated to the low-quality reward.

### Food preference test

In a previous experiment with the same subjects, walnut had been used as high-quality reward and sunflower seed as low-quality reward (Krasheninnikova et al. 2018). For the present study, a preference test established whether the subjects still preferred walnuts over sunflower seeds. To this end, each reward was placed on the respective individually designated food holder colour (see description above), before presenting both food holders to the subject simultaneously (see Table 1 for an overview).

We performed 12 trials, with the position of the coloured food holders pseudo-randomised. To reach criterion, the subject had to choose walnut at least nine times out of the 12 trials (*p* = 0.019 one-sided binominal test). All 28 tested individuals reached this criterion (including the exception of one individual who was not tested again by mistake after reaching only 8/12) within one test session, thus confirming a clear preference for walnut.

### Habituation

A habituation phase with forced trials was implemented to habituate the subjects to the apparatus and the rotation of the food holders (i.e. movement, noise), as well as to familiarise them with the procedure (see Table 1 for an overview). Only a single food holder attached to the upper wooden disk was presented in a position out of the subject’s reach. During the whole process, the apparatus was held in a retracted position, preventing the subject to reach the reward before it reached the end position. When the reward was in front of the opening between the chambers, the motor was turned off and the apparatus was pushed within reach of the subject. The test consisted of 12 trials counterbalancing the reward type across trials (i.e., six trials low-quality reward, six trials high-quality reward) in pseudo-random order. If a subject took each of the 12 rewards within 30 s without showing any signs of fear (i.e. readily approaching the apparatus without hesitation), it had reached criterion and proceeded to the test. If a bird did not take the reward within 30 s, the trial was repeated; however, if the bird did not respond in three consecutive trials, the session was terminated and repeated on the next day. The majority of birds reached this criterion within two sessions while one bird needed four sessions.

### Delay of gratification test

#### Experimental procedure

The procedure was the same as in the previous habituation except that two food holders were now present simultaneously and the apparatus was in the forward position from the start.

**Fig. 2:**
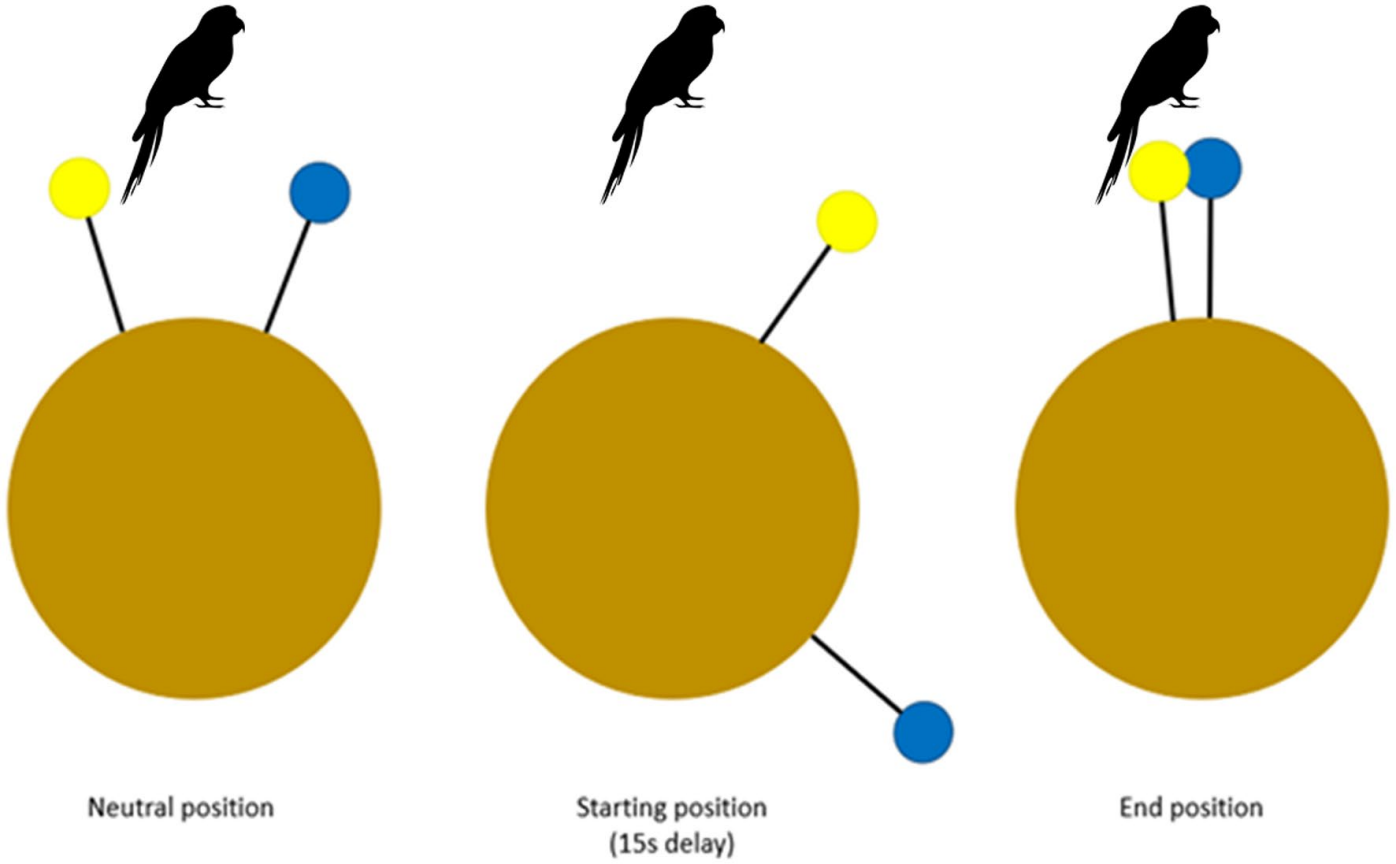
Schematic representation of the food holders’ positions relative to the parrot. In this example, the HQR is placed in the blue food holder, the LQR in the yellow one. Food holders were placed in the neutral position at the beginning of each trial, while showing the rewards to the subjects. Then, food holders were moved to the starting position, with the LQR always placed at the fixed LQR starting position (i.e. arriving in its LQR end position in front of the bird after 5 s) and the HQR placed at the respective delay (in this example, the HQR comes after 15 s). In each trial, food holders reached the end position despite the individual choice (if the LQR was selected, the apparatus was pulled out of reach, but the motor never stopped until the HQR food holder reached its end position)

During the presentation of the reward types at the beginning of each trial, the two food holders on the apparatus were arranged in a neutral position (both food holders in front of the window, ca. 20 cm apart from each other, see Figs. 1b and 2). Next, the rewards were put on the corresponding food holders and the disks were arranged into their respective starting position. Once the rewards were placed on the food holders, the engine was turned on via a switch controller. To ensure that the parrots were attending to the overall setup in each test condition rather than simply grabbing an immediately available reward, the low-quality reward reached the bird only after 5 s and then remained in this position for the remaining time. If the subject chose the low-quality reward, the experimenter immediately retracted the apparatus, so the food holders moved out of reach. The engine kept running until the high-quality reward food holder arrived in the end position (in front of the subject near the low-quality reward food holder, see Fig. 2), which the subject could see but not reach anymore. If the bird, however, refrained from choosing the available low-quality reward, the apparatus remained in the forward position until the high-quality reward food holder moved into reach. After the bird took the high-quality reward, the apparatus was immediately retracted, preventing the subject from picking up the second reward, and the trial ended. After an inter-trial interval of 30 s, during which the experimenter adjusted or changed the food holders and reset the apparatus, the next trial started.

#### Experimental conditions

One test session consisted of four *demonstration* and 14 test trials. The test trials consisted of ten *delay trials*, two *low-quality reward controls*, two *position controls*. Each session started with the demonstration trials followed by the test trials in random order (see Table 1 for an overview).

The purpose of the *demonstration trials* was to inform the birds about the delay duration of the upcoming *test trials* and to ensure that they remained familiar with the procedure. During *demonstration trials*, the apparatus was presented in the retracted position, so that the birds could only passively watch the rotating food holders until the respective delay (which was the same as that of subsequent test trials) had passed. Only once both food holders had reached the end position, the apparatus was pushed forward so the bird could choose a reward (see Video S1). To pass the *demonstration trials*, the subject had to choose the high-quality reward in all four trials. As soon as it picked up the high-quality reward, the apparatus was retracted, preventing the subject from picking up the second reward. All subjects always selected the high-quality reward in the demonstration trials.

*Test trials* differed from demonstration trials, in that the apparatus was presented in the forward position, thus within reach of the bird, from the beginning of the trial, so that the subject could decide freely whether to wait for the second food holder to move within reach or not (see Video S1).

In the critical tests, i.e. the *delay trials*, the bird faced a choice between an immediately available low-quality reward, and a high-quality reward, available after a certain delay (minimum of 5 s- maximum of 60 s after the low-quality reward reached its end position). Birds were considered successful if they refrained from eating the low-quality reward reward and instead waited for high-quality reward to become available. A failure was coded instead, if the bird consumed the low-quality reward before the high-quality reward had moved within reach. The delay between low-quality reward and high-quality reward was gradually increased, depending on the individual’s success, starting from 5 to 10 s, 15 s, 20 s, 25 s, 30 s, 40 s, 50 s and up to the maximum of 60 s. To proceed to the next delay stage, the subject had to successfully wait for the high-quality reward in at least four out of ten delay trials in two consecutive sessions. If a bird did not reach this criterion within six sessions, the test was terminated. If a parrot reached criterion in the sixth session, a session with a correspondingly longer delay was conducted.

To verify whether the birds indeed waited for the better reward and actually paid attention to the reward contingencies, rather than just applying a learned rule, e.g., to avoid the low-quality reward food holder or to wait for the second option, the *low-quality reward control* and the *position control* were implemented. In the *low-quality reward control*, the birds were presented with two food holders of the same colour both of which carried low-quality reward (which of the two food holder colours corresponded to low-quality reward differed across birds pseudo-randomly, see description above). The set-up and overall procedure were the same as in *delay trials*: the food holder on the upper disk moved into reach after 5 s, while the food holder on the lower disk became available after the same delay as in the corresponding *delay trials*. Again, the birds could choose between the two options; however, as both food holders contained the same reward type, waiting for the second option involved an unnecessary cost rather than bringing about a benefit. If the subjects understood the contingencies and were capable of deciding optimally rather than following learned rules, they should choose the first low-quality reward.

In the *position control*, the position of the food holders was switched so that the food holder carrying the high-quality reward was now arriving first while the low-quality reward food holder became available after the delay. The colours of the food holders remained allocated to the same reward type for each subject as before, and also the general setup and procedure did not differ from the delay trials. While theoretically, birds could fail the *low-quality reward control* due to lack of attention to the second food holder (i.e. not noticing that it is now low-quality reward instead of high-quality reward as in delay trials), the state of affairs is even more striking in the *position control*, where the high-quality reward is available immediately. Here, it should be obvious that it does not pay off to wait any longer even if the birds do not take note of the second food holder that only contains low-quality reward. Thus, if the birds understand the contingencies of the task and make economic decisions, rather than just sticking to learned rules, they should not wait for the second reward, which is now not only delayed but also less preferred.

### Analyses

#### Behavioural coding

The videos were coded using Solomon Coder (2015 by András Péter). For the *delay trials* and *control conditions*, we coded the subject’s choice and the latency between starting the motor and picking up a reward (high-quality reward or low-quality reward). Only for the *delay trials* condition, we analysed the birds’ behaviour during each trial. We coded the duration of (i) object manipulation behaviours (i.e. biting the perch, the table or the plexiglass, playing with their metal ring, and manipulating old seed husks), (ii) locomotor behaviours (i.e. pacing about in the experimental chamber or sitting on the perch), (iii) body position (i.e. time spent facing or turned away from the apparatus/reward), and (iv) distance to rewards (i.e. time spent on the front or back half of the table). A second coder analysed 20% of the videos for reliability. Cohen's kappa for individual choices in test and control conditions was = 1. The intraclass correlation coefficient (3) for all the behavioural variables coded during each trial was > 0.7.

### Statistical analyses

All analyses were conducted in R (v. 3.5.3) using the ordinal (v2019.12-10) (Christensen 2015), and MCMCglmm (v. 2.29) (Hadfield 2010) packages. In a post hoc examination of the duration between when the high-quality reward was available to the subject and when the subject retrieved the high-quality reward showed large variation (minimum = 0.200, mean = 3.873, maximum = 54.000). The larger durations may suggest that the subjects were not attentive to the apparatus and were, thus, too distracted during the trial or were not motivated to attend to the rewards. To reduce potential bias towards higher maximum delays, we left out the top quartile of positive durations (> 4.6 s difference between high-quality reward arrival and high-quality reward retrieval). This reduced our trial number from 4590 to 3790.

#### Species maximum delay

To understand species differences in maximum delay, we fit a Bayesian model with an ordinal distribution of maximum delay as a function of sex and species. An interaction between sex and species was included but was non-significant and therefore removed. Sex as a main effect was included in the analysis as a control for any potential differences between males and females. Individuals that did not pass the 5 s delay were given a maximum delay of 0. We used a prior with residual variance fixed to 1. To verify our results, we also conducted a cumulative link model with the same explanatory variables. This provided qualitatively similar results and we therefore only present the Bayesian analysis here. Bayesian analysis uses probability for quantifying uncertainty of parameters based upon our data (Gelman et al. 2013; McElreath 2016). Thus, we can use our prior knowledge of a system and our data to obtain the relative credibility of parameters.

#### Individual success within a session

We also wanted to understand how individual characteristics influence overall success within a session. We define success as whether a subject waited for and took the high-quality reward. Failures were trials where the low-quality reward were chosen. We fit a Bayesian mixed effects model using the family “multinomial2” with success and failures as a function of trial number, delay time as a factor, the sum in seconds of all object manipulation behaviours, locomotion behaviours, the amount of time at the back half of the table (away from the experimental setup), body oriented away from the apparatus, and other behaviours that may indicate distraction or coping into one larger “coping” variable. We corrected for this coping variable by the summed test duration of the session. We also included sex, species, and residual weight. The coping variable and trial number were scaled to a mean of 0 and a standard deviation of 1. We included residual weight as a proxy for motivation to wait for the high-quality reward. We calculated these estimates by fitting a linear model of weight as a function of day for each individual. We then extracted the residual for each data point. Thus, a negative residual would be associated with a hungry bird that might not wait for a high-quality reward. Individual was also included as a random effect. Because we were interested in how coping behaviour influenced success as delays increased and among species, we also included an interaction between the coping variable and delay time and an interaction between coping and species. Individual and session number were included as random effects. In our final analysis of coping time and delay, we set our delay time reference to 10 s delay after exploratory analysis suggested that this time unit may be more biologically meaningful and therefore easier to interpret. A model with five second delay as the reference group resulted in negative coefficients for the interaction as delay time got larger. This is most likely because at five seconds, individuals that performed relatively little coping behaviours were still able to be successful in the task. Thus, as time delays got larger, individuals with relatively higher proportion of coping time were still less successful than lower coping individuals at the five second delay.

To investigate if the parrots can anticipate the upcoming delay duration and give up waiting early on during a non-successful trial, we analysed the latency to pick up the low-quality reward (“giving up times”) during each trial and compared it to a constant chance of giving up (e.g. as in Dufour et al. 2007). Using a Kaplan–Meier survival analysis, we entered successful and unsuccessful trials as censored data and calculated the probability of each bird to wait longer than the already elapsed time. This observed distribution of giving up times was compared to an expected chance of giving up (i.e. constant chance of giving up throughout each trial). The observed and expected distributions of giving up times were compared using a Kolmogorov–Smirnov test (Haccou and Meelis 1992).

Finally, we wanted to test for the effect of distinct coping elements by conducting a separate Bayesian mixed effect model of success within a session as a function of each coping component. This analysis included a subset of the coping elements used in the previous analysis above because several had a variance inflation factor above 3. Our final analysis included pacing, manipulating the ring, perch, table, door, Plexiglas, empty seed husks, and biting the arm of the food holder. We corrected for duration of trials during the session, and all variables were scaled to a mean of 0 and a standard deviation of one. We also included individual as a random effect.

All mixed effects models used an inverse Wishart and expanded priors with random effects as *V* = 1, nu = 0.002, alpha.mu = 0 and alpha.*V* = 1000 and residual variance as *V* = 1 and nu = 0.002 (Hadfield 2010). Each model had a thinning interval of 10,000, burnin = 500,000, and was run for 5,000,000 iterations. All model trace plots were checked visually, and autocorrelation was less than 0.15. An inverse Wishart prior is a relatively uninformative prior that describes a multivariate normal distribution, and the expanded priors help with sampling and convergence with variances close to 0. The large number of iterations were used to help obtaining large enough effective sample sizes.

## Results

### Preference tests

Five out of the 28 parrots exhibited a preference for one of the two coloured arms (i.e., two for blue and three for yellow) and, consequently, were assigned the opposite colour as their high-quality reward colour. All other parrots exhibited no preference for either colour (see Supplementary material). In the food preference test, we found that consistently all parrots preferred the walnut over the sunflower seed (see Supplementary material).

### Control trials

All four parrot species significantly preferred the first option during the position control, in which the high-quality reward was delivered first (AGP: 1.98 ± 0.16, BHM: 2.00 ± 0.00, BTM: 1.99 ± 0.12, GGM: 2.00 ± 0.00 successful out of two trials), and likewise selected the first option in the low-quality reward control, in which both arms contained the low-quality reward (AGP: 1.94 ± 0.31, BHM: 1.83 ± 0.49, BTM: 1.77 ± 0.48, GGM: 1.85 ± 0.37 successful out of two trials). Success in the low-quality reward control trials increased across test sessions (LM: 0.037 ± 0.012, *t* = 3.135, *p* = 0.002), while success in the position control trials remained constant across test sessions (LM: − 0.001 ± 0.001, *t* =  − 0.902, *p* = 0.367).

### Delay trials

#### Species maximum delay

**Fig. 3:**
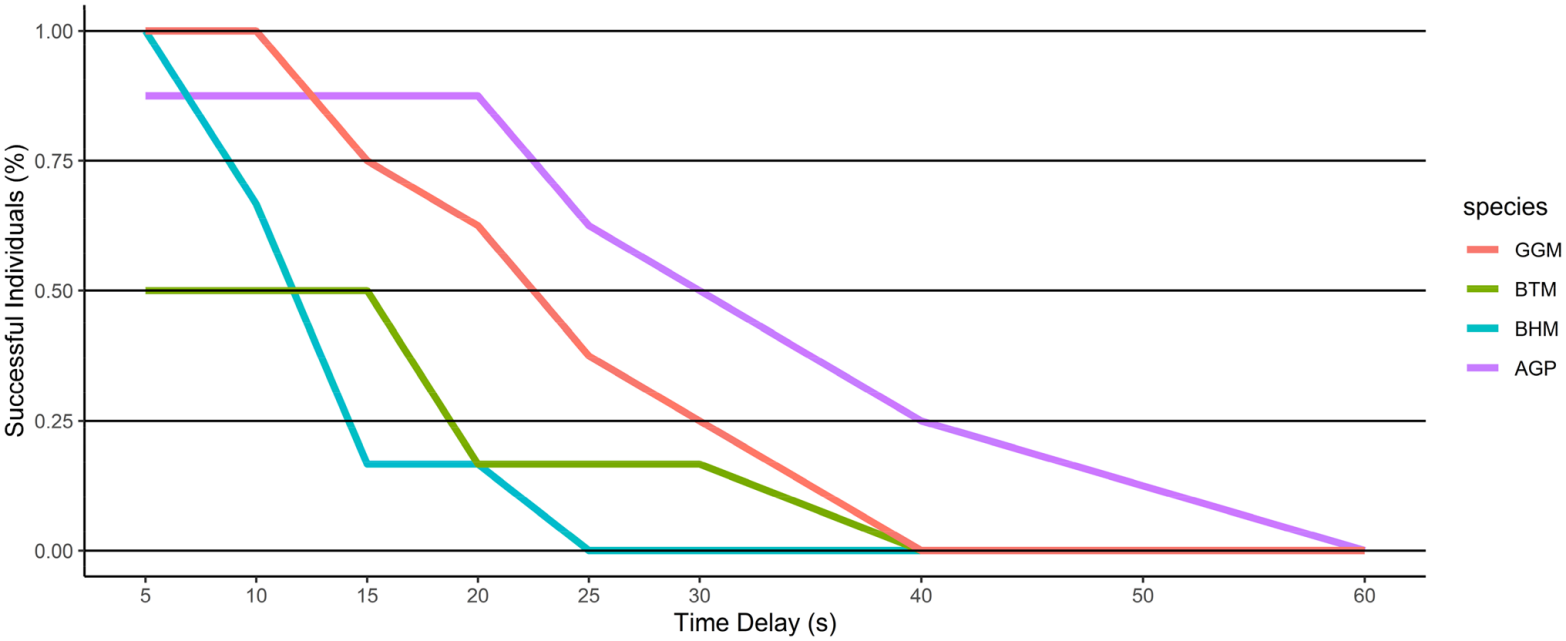
Percentage of successful individuals per delay stage. The different species are plotted separately (*GGM* great green macaws, *BTM* blue-throated macaws, *BHM* blue-headed macaws, *AGP* African grey parrots)

The mean and maximum delay the subjects were waiting for the high-quality reward are presented for each species in Table 2. The percentage of individuals per species achieving the increasing delay steps are shown in Fig. 3 (see also Fig. S1). African grey parrots achieved higher maximum overall waiting durations (= “delays”) (see Table 2 and Fig. 3 for an overview and Table 3 for statistical comparisons) than all three macaw species–great green (posterior mean = 1.622; 95% CI 0.235–3.177; pMCMC = 0.013), blue-throated (posterior mean = 2.2851; 95% CI 0.243–3.904; pMCMC = 0.031) and blue-headed macaws (posterior mean = 2.165; 95% CI 0.281–3.742; pMCMC = 0.018). Males had overall lower maximum delays than females (Table 2).

**Table 2:** Mean and standard deviation of waiting performance for each species and the maximum waiting duration (= delay) for an individual within that species

Species	Max. delay: group-level (mean ± SD)	Max. delay: individual-level
Great green macaws (GGM)	20.00 ± 8.02 s	30 s
Blue-throated macaws (BTM)	8.33 ± 11.70 s	30 s
Blue-headed macaws (BHM)	11.70 ± 6.06 s	20 s
African grey parrots (AGP)	29.40 ± 15.20 s	50 s

**Table 3:** Posterior mean, 95% lower and upper credible intervals (CI), and estimated pMCMC values for sex and species during the maximum delay (= waiting time)

Variable	Posterior mean estimate	95% lower CI	95% upper CI	pMCMC
(Intercept)	2.881	1.454	4.228	0.002
**Sex (male)**	**– 1.519**	**– 3.060**	**– 0.188**	**0.036**
Blue-throated macaw	– 1.133	– 2.910	0.802	0.236
Blue-headed macaw	– 0.588	– 2.215	1.032	0.511
**African grey parrot**	**1.622**	**0.235**	**3.177**	**0.013**

Females and the great green macaw are the reference group

Significant effects, those variables with 95% CI excluding 0, are highlighted in bold

#### Individual success within a session

**Fig. 4:**
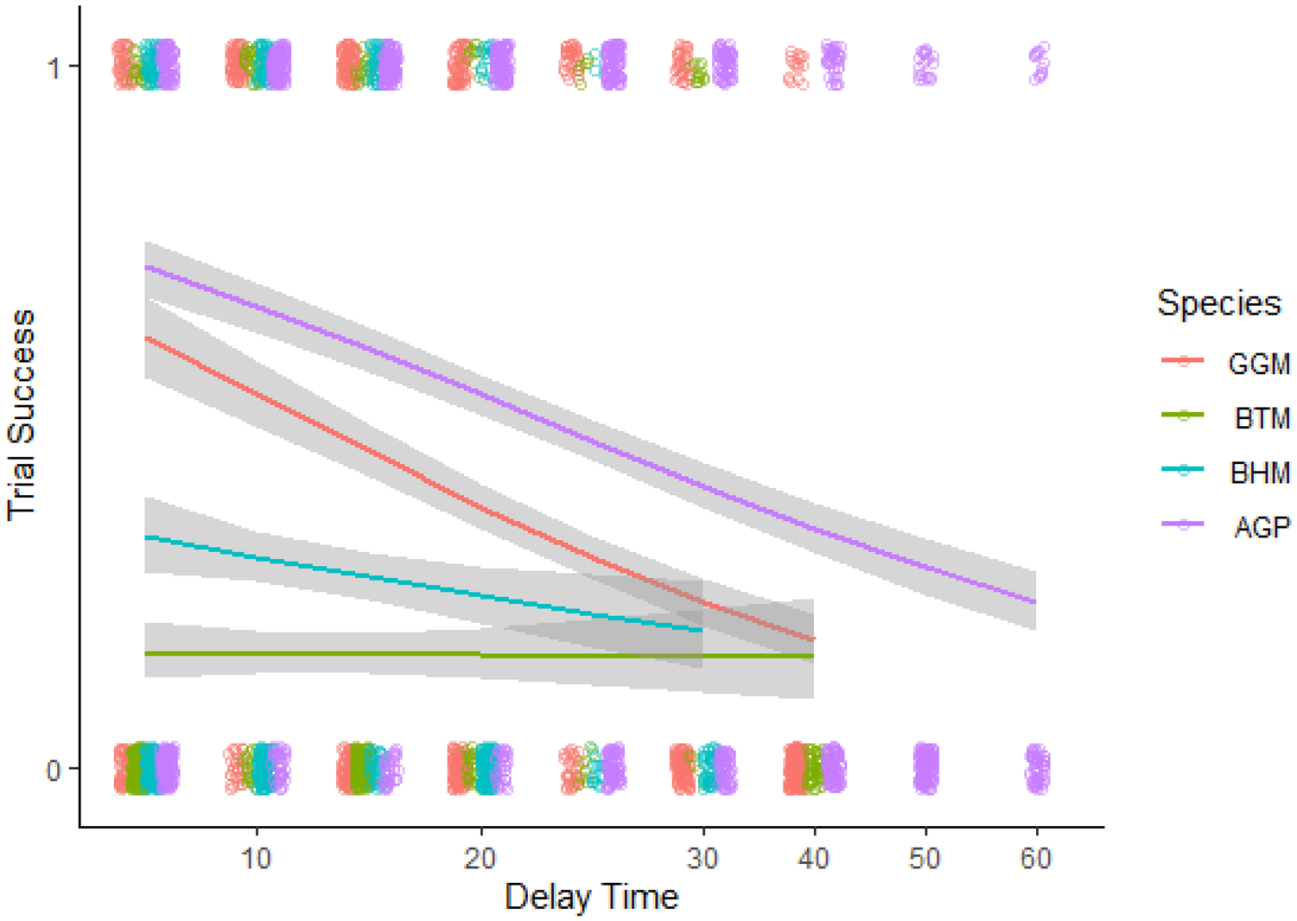
The effect of delay on probability of an individual successfully waited for a HQR across the four species. Grey ribbons are 95% confidence intervals

We found that species differed in their success (i.e. choosing the high-quality reward) (Table 4; Fig. 4), and that this interacted with coping time. Generally, individuals did worse as delay time increased. Furthermore, the effect of coping behaviour shows that individuals spending more time engaging in coping behaviours achieved higher success, and this effect interacted with delay time so that individuals that had higher coping times were generally more successful as delays increased when compared to the 10 s delay (Table 4). Furthermore, comparisons at the species level show that the great green macaws that had higher proportion of time using coping behaviours had higher success than the blue-throated macaw with the same proportion of time using coping behaviours (Table 4). African grey parrots had greater success than both the blue-throated macaws and the blue-headed macaws with the same proportion of time coping (AGP-BTM posterior mean = 0.324; 95% CI 0.129–0.524; pMCMC < 0.002; AGP-BHM posterior mean = 0.174; 95% CI 0.010–0.330; pMCMC = 0.036). We found no difference between blue-throated macaws and blue-headed macaws that had similar proportion of time coping (BTM-BHM posterior mean = 0.148; 95% CI  − 0.040–0.340; pMCMC = 0.120). We found no association between trial number and residual of body weight and success to wait for the high-quality reward. We also found no differences between the sexes (Table 4).

**Table 4:** Posterior mean, 95% lower and upper credible intervals (CI), and estimated pMCMC values for variables included in our model of success

Variable	Posterior mean estimate	95% lower CI	95% upper CI	pMCMC
**(Intercept)**	**– 2.886**	**– 3.609**	**– 2.204**	**0.002**
**Time delay 5**	**– 0.199**	**– 0.382**	**0.026**	**0.076**
**Time delay 15**	**– 0.222**	**– 0.416**	**– 0.049**	**0.013**
**Time delay 20**	**– 0.657**	**– 0.875**	**– 0.463**	**0.002**
**Time delay 25**	**– 0.603**	**– 0.841**	**– 0.366**	**0.002**
**Time delay 30**	**– 0.905**	**– 1.147**	**– 0.621**	**0.002**
**Time delay 40**	**– 1.855**	**– 2.181**	**– 1.483**	**0.002**
**Time delay 50**	**– 2.347**	**– 2.875**	**– 1.720**	**0.002**
**Time delay 60**	**– 1.596**	**– 2.220**	**– 0.893**	**0.002**
**Coping**	**0.245**	**0.065**	**0.393**	**0.013**
Sex (M)	– 0.391	– 0.974	0.405	0.227
**Blue-throated macaw**	**– 1.082**	**– 1.942**	**– 0.131**	**0.027**
Blue-headed macaw	– 0.424	– 1.221	0.331	0.249
African grey parrot	0.235	– 0.486	0.876	0.453
Residual weight (g)	0.002	– 0.001	0.004	0.284
Trial number	0.012	– 0.048	0.075	0.684
Time delay 5 * coping	**0.551**	**0.327**	**0.735**	**0.002**
Time delay 15 * coping	**0.218**	**0.051**	**0.357**	**0.004**
Time delay 20 * coping	**0.321**	**0.183**	**0.495**	**0.002**
Time delay 25 * coping	**0.278**	**0.085**	**0.443**	**0.004**
Time delay 30 * coping	**0.491**	**0.286**	**0.733**	**0.002**
Time delay 40 * coping	**0.272**	**0.042**	**0.455**	**0.004**
Time delay 50 * coping	**1.002**	**0.564**	**1.311**	**0.002**
Time delay 60 * coping	0.217	– 0.104	0.501	0.182
Blue-throated macaw * coping	**– 0.255**	**– 0.430**	**– 0.032**	**0.009**
Blue-headed macaw * coping	– 0.101	– 0.272	0.065	0.204
African grey parrot * coping	0.066	– 0.091	0.243	0.476

We used females, the great green macaw, and the 10 s time delay as reference groups

Significant effects, those variables with 95% CI excluding 0, are highlighted in bold

#### Coping behaviour

In our model of coping behaviours, several distractive behaviours had a positive effect on success within a trial with pacing having the largest effect (Table 5).

**Table 5:** Posterior mean, 95% lower and upper credible intervals (CI), and pMCMC for coping behaviours included in our model of success in trials

Variable	Posterior mean estimate	95% lower CI	95% upper CI	pMCMC
**(Intercept)**	**– 3.692**	**– 3.968**	**– 3.336**	**0.002**
**Pacing**	**0.594**	**0.533**	**0.644**	**0.002**
Ring	0.060	0.009	0.120	0.053
Perch	0.017	– 0.036	0.061	0.471
**Table**	**0.174**	**0.127**	**0.226**	**0.002**
Door	0.028	– 0.015	0.072	0.240
**Bite Arm**	**0.090**	**0.038**	**0.139**	**0.002**
**Plexiglass**	**0.096**	**0.051**	**0.142**	**0.002**
Seeds	0.036	– 0.013	0.090	0.182

Significant effects, those variables with 95% CI excluding 0, are highlighted in bold

#### Giving up times

We found that all individual parrots gave up waiting earlier than expected by a constant “giving up chance” (see Supplementary Material). During trials in which the birds did not wait for the high-quality reward, they consumed the low-quality reward after several seconds rather than later on during the trial.

## Discussion

Our investigation of self-control using a delay of gratification paradigm revealed that all species (great green macaw, blue-throated macaw, blue-headed macaw, and African grey parrot) were able to wait for the better reward. However, there were substantial species variation. In particular, the African grey parrots tolerated higher delay times than the macaws. The mean group level waiting time for the African grey parrots equaled the highest waiting time achieved by two macaw individuals. Furthermore, an individual grey parrot waited a maximum of 50 s. Among the three macaw species, the great green macaws and blue-throated macaws reached a maximum delay of 30 s, and the blue-headed macaw did not wait longer than 20 s for the high-quality reward. We also found an interesting sex effect with females having longer maximum delays than males. Individuals that distracted themselves while waiting for the high-quality reward were more successful than individuals that just waited passively. Within a session, we found no effect of sex or residual body weight on the number of successful trials. Overall, if parrots gave up during a trial, they did so early on instead of at an arbitrary point; thus, suggesting that they were able to anticipate the delay duration.

We observed substantial variation in the capacity to delay gratification among the four species as well as success within a session. Notably, the blue-throated macaw, was the least patient species in terms of the minimum threshold they were prepared to wait. Only three out of six individuals (50%) of the blue-throated macaws passed even the 5 s delay stage, whereas 100% of the great green macaw, 100% of the blue-headed macaws, and 88% (seven out of eight) of the African greys readily mastered this short duration. Three of these successful blue-throated macaws reached a max. delay of 10 s, while one waited for 30 s; thus, outperformed the blue-headed macaws in terms of individual but also group level performance. In controlling for individual motivation by including the residual of the daily body weight in the analyses of success within a session, we could rule out that different levels of motivation or hunger were affecting the observed performance in the test.

As expected, with increasing delays, the individual success rate dropped and the species-specific maximum delays were reached only by a single individual; nonetheless, also on a group-level African grey parrots excelled. Another study has reported that one African grey delayed gratification for up to 900 s (Koepke et al. 2015), although, the bird was trained to respond to a verbal “wait” command in a very different, training-intense paradigm; thus, rendering the direct comparison difficult. The only other study that assessed African grey parrots’ self-control abilities tested them in an accumulation task, in which the three subjects failed to wait for longer than 3 s (Vick et al. 2010). Nonetheless, compared to other parrot species that have been tested in exchange-based paradigms, like Goffin cockatoos [max. 80 s (Auersperg et al. 2013)] and kea (max. 160 s (Schwing et al. 2017)), the macaws performed modestly (max. 30 s). Whether this is indeed an indication for reduced self-control capacities in the Arinae subfamily compared to the cockatoos and New Zealand parrots, however, remains unanswered as performance in different delay of gratification paradigms might not be comparable (Miller et al. 2019a; Susini et al. 2020).

In line with this, we have tested these same individuals in two previous studies that assessed other forms of behavioural inhibition and obtained contradicting results. First, we tested them in the cylinder task (Kabadayi et al. 2018), which was meant to measure their motor inhibition and resulted in very poor performance compared to corvids and primates (Kabadayi et al. 2017). In this previous study, the great green macaws outperformed the other three parrot species, whilst the blue-headed macaws and African greys performed the poorest, but the task likely did not measure the parrots’ actual motor inhibition ability and therefore should not be used further in parrots (Kabadayi et al. 2017; see also von Horik et al. 2018). It is an example lending support to the claim that different measures for behavioural inhibition often do not measure the same behavioural construct (Tsukayama and Duckworth 2010; Bray et al. 2013; Brucks et al. 2017a; von Horik et al. 2018; Vernouillet et al. 2018). Second, we showed that in an exchange task paradigm, macaws and African greys could differentiate between a food item and a token representing a higher-quality food item in order to assess their ability to maximise their payoff (Krasheninnikova et al. 2018). All species significantly selected the token despite the value of the immediate reward (low vs medium quality enhance), showing the ability to forgo an immediate reward to maximise the payoff, however, without enduring a delay; thus, suggesting that all four species show the basic capacity for making correct decision in costly situations.

We found that females reached higher maximum delay times than males when controlling for species differences. While it needs to be noted that the sample size was not perfectly balanced for sex (11 M/17F), linear mixed effects models are capable of handling unbalanced designs, and this finding still is of interest as other studies on behavioural inhibition did not find any sex effects (Brucks et al. 2018; Miller et al. 2019b; van Horik et al. 2018). On the contrary, some studies detected marked differences in males’ and females’ inhibition abilities. For example, male pheasants show better behavioural inhibition than females. Van Horik et al. (2017) found that male pheasants participate more consistently in cognitive tests, which involve food rewards, compared to females. This might be due to males being more food motivated and/or bold (van Horik et al. 2017). Meier et al. (2017) supported these results showing that male pheasants exhibited greater inhibition in a novel response-inhibition task. Other studies in fish found the opposite pattern as females outperformed males in a motor inhibition task (Brandão et al. 2019). Future studies need to investigate if species-specific roles in food acquisition and provisioning to the mate or offspring might govern these differences.

Certain behaviours exhibited during the delay period related to an individual’s waiting success. In particular, we observed several types of behavioural categories: object manipulation behaviours, such as biting the table, perch, ring on their leg or Plexiglas, and locomotory behaviours, such as pacing, and staying away from the apparatus. We found that these behaviours seemed to enhance the waiting performance overall, with individuals exhibiting these behaviours for proportionally longer durations succeeding in more trials than individuals that distract themselves less. Furthermore, coping behaviours were gradually more important for success with increasing delay durations when compared to the 10 s delay. This raises the possibility that these behaviours might indeed help the subject to divert its attention from the appeal of the available food piece while waiting for the better option and/or to cope with suppressing the constant conflicting impulse to take this available food right away. Whether these individuals, however, divert their attention intentionally remains speculation. We also found that longer coping behaviour duration benefited some species more than others. African greys that performed coping behaviours did better than some of the other species that spent a similar amount of time engaged in coping behaviours.

Interestingly, similar coping behaviours have been reported also in many other species, and likewise were indicative of an enhanced waiting performance. Dogs and wolves, for example, distracted themselves with distancing themselves from the available low-quality reward, laying down and looking away (Leonardi et al. 2012; Brucks et al. 2017b; Range et al. 2020). Chimpanzees tolerated higher delays, if they could interact with toys during the delay (Evans and Beran 2007b) and children waited longer, if they rested their head on their arms and closed their eyes (Steelandt et al. 2012). Even though no study in birds has directly investigated the effect of displaying specific coping behaviours on self-control abilities, many studies reported that the birds exhibited characteristic behavioural patterns while waiting. Ravens and crows were pacing and caching the food rewards during the delay duration (Dufour et al. 2012) and New Caledonian crows visually tracked the food reward and increased the distance to the available low-quality reward while waiting (Miller et al. 2019b). Cockatoos exhibited food-directed behaviours towards the low-quality reward, which they needed to hold while waiting (i.e. manipulating the reward) but also showed stereotypic locomotor patterns (i.e. pacing, turning, swaying; (Auersperg et al. 2013)). The African grey in Koepke et al.’s (2015) study distracted himself with preening, looking away or manipulating the reward while waiting. In our study, pacing the experimental chamber was the coping behaviour that had the greatest effect on success across all species, maybe because it also involved looking away and distancing themselves from the reward.

In addition, it is noteworthy that all parrots seemed capable of anticipating the delay duration. In unsuccessful trials, the parrots gave up waiting (and took the low-quality reward) at the beginning of a trial instead of waiting for some time before giving up at an arbitrary time point during the delay duration. Likewise, kea (Schwing et al. 2017), cockatoos (Auersperg et al. 2013), ravens and crows (Dufour et al. 2012) could anticipate delay durations; thus, suggesting that birds have an understanding of upcoming time durations.

Our results suggest stark differences between species in their self-control abilities. Several hypotheses have been raised over recent years trying to explain species these differences including foraging ecology, metabolic rate, (relative) brain size, and social complexity. We discuss them here in view of our findings on the premise that we can only draw very tentative conclusions. Stevens et al. (2005) suggested that the feeding ecology of a species is linked to its self-control abilities. Accordingly, extractive foragers or species that need to wait for resources to become available (e.g. gummivorous feeders) may possess better self-control abilities than species, which can quickly assess resources, such as insectivorous species. While the feeding ecology of the tested parrot species is only fragmentarily known, the two bigger macaw species (GGM, BTM) can be considered specialists, as great green macaws heavy rely on seeds of almond mountain trees (Berg et al. 2007) and blue-throated macaws on the mesocarp of the motaçu palm fruit (Yamashita and Machado de Barros 1997). African grey parrots and blue-headed macaws, on the contrary, can be considered generalists since they feed from multiple resources and have a rather granivorous diet (Juniper and Parr 1998; Tobias and Brightsmith 2007). This hypothesis, however, cannot fully explain our results as the African greys as generalists outperformed the macaw species. Amongst the macaws, the specialist great green macaws performed best, however, the generalist blue-headed macaws were better than the specialist blue-throated macaws. Potentially, other or more specific factors of the feeding ecology (i.e. proportion of diet, time invested into locating/extracting food) are better predictors for a species’ self-control capacity than the general dietary breath (see also van Horik et al. 2018).

Other hypotheses are based on morphological aspects, such as body size and metabolic rate. Metabolic rate, as estimated by body mass, has been proposed to predict self-control abilities, because smaller species with a high metabolic rate need to replenish their energy supplies more frequently and therefore cannot afford to wait (Stevens and Stephens 2010). They should show reduced self-control abilities compared to species with low metabolic rates. Our data does not support this hypothesis, as the African grey parrots with 450 g of body weight outperform the almost three times bigger great green macaws (1300 g).

Other hypotheses try to explain self-control abilities based on brain size or social complexity, however, given the incomplete knowledge about the brain size and social organisation of less intensively studied species that are threatened by extinction in the wild, such as the blue-headed macaws and the blue-throated macaws, we can only make speculative guesses. Species with bigger brains (in terms of both absolute brain size and relative brain size) have been hypothesised to possess enhanced general cognitive capacities, including behavioural inhibition (MacLean et al. 2014). While absolute brain size was found to be a predictor in a comparative dataset predominately consisting of primate species (MacLean et al 2014), relative brain size was a better predictor if the focus was on corvids (Kabadayi et al. 2016). According to their relative brain size, the African greys should possess better self-control abilities (relative brain size: 0.023; Iwaniuk et al. 2005; Olkowicz et al. 2016) than the great green macaws (relative brain size: 0.013; Iwaniuk et al. 2005; Olkowicz et al. 2016), while data on the brain size of the two other macaw species is not available. Our finding that the African grey parrots performed best supports this hypothesis but does not allow us to draw strong conclusions. A further hypothesis proposes that behavioural inhibition is linked to social complexity. Accordingly, species living in more complex social environments (e.g. fission–fusion societies) require more behavioural inhibition than species living in simpler social organisations because they need to inhibit their actions more often (Amici et al. 2008). At least in terms of group size and because they exhibit fission–fusion dynamics during social foraging in the wild (Dändliker 1992), African grey parrots may be assumed to exhibit the most complex social organisation of the species we examined. The macaw species appear to exhibit simpler social organisations compared to the African grey parrots, at least in terms of flock size and cohesiveness. The great green macaws might be considered the least gregarious species with only up to nine individuals within a flock (Berg et al. 2007), while the blue-throated macaws (Yamashita and Machado de Barros 1997) and blue-headed macaws (Tobias and Brightsmith 2007) have been observed in flocks of up to 15 and 60 individuals in the wild. Nonetheless, these numbers need to be treated with caution, as all three macaw species only remain with very low population numbers in the wild and also because flock size is not necessarily indicative of social complexity (Emery et al. 2007). Again, our results only partially support this hypothesis, as African greys, which do live in complex fission–fusion societies, outperformed the other macaw species, which potentially live in smaller and less complex social groups. However, among the macaws the reported group sizes did not seem to relate to self-control and much more comparative data would be necessary for drawing convincing conclusions. Overall, it needs to be acknowledged that we cannot disentangle phylogeny and social complexity as the African grey parrots differ in both of these aspects from the macaws.

The rotating tray task proved to be a useful tool for assessing self-control abilities of parrots as it avoids the challenges for parrots associated with the exchange task, namely having to keep the low-quality reward directly in the beak (thus their taste organ) throughout the waiting period. Yet, contrary to previous studies, we implemented an important change, which made the rotating tray task more comparable to other delay of gratification paradigms, particularly the exchange task, as the low-quality reward remained accessible throughout the whole delay duration. In previous studies (Bramlett et al. 2012; Perdue et al. 2015), the low-quality reward food holder passed the individual and rotated out of reach after a certain time, while the high-quality reward food holder was not available yet. Consequently, once the low-quality reward food holder had passed subjects were forced to wait the remaining time, rather than having the opportunity to “give up” waiting at any point in time prior to the arrival of the high-quality reward.

We believe the rotating tray task with our modification could be a universally applicable tool for measuring self-control comparatively for the following reasons. Foremost, it requires no direct contact with the subjects (e.g. in the exchange task where subjects need to be trained to exchange items with an experimenter) and thus, it is also applicable to less habituated animals. On the other extreme, it also has clear advantages for testing species that are highly sensitive to experimenter cues (e.g. domesticated species) and that could be inadvertently cued by the experimenter. Furthermore, it is an intuitive task that requires little training as opposed to, for example, the exchange task, thus, reduces the drop-out rate due to failures in reaching training criteria. This increases the validity of results, as also poorly performing individuals that would have dropped out in training are included, which might account for the substantial individual variation in self-control abilities encountered in this study. Future research needs to validate the rotating tray task by (1) assessing how our modifications affect the performance (i.e. whether indeed the modified version is more difficult) and (2) test how the performance in the modified rotating tray task correlates with performance in other delay of gratification paradigms, such the widely used exchange task (Brucks et al. 2017a).

## Conclusion

In conclusion, we show that the great green macaw, blue-throated macaw, blue-headed macaw, and the African grey parrot of the parrot superfamily Psittacoidea can delay gratification with African grey parrots (subfamily Psittacinae) clearly outperforming the three macaw species (subfamily Arinae) displaying the highest maximum delay and a two to three times higher maximum delay on a group level. The blue-throated macaws and great green macaws reached similar maximum delays, yet the great green macaws performed better on a group level. Blue-headed macaws had the lowest individual and group-level maximum delay. Furthermore, individual coping strategies (e.g. pacing) during the delay time affected waiting performance positively across all four species. Socio-ecological factors but also brain size or general intelligence might be drivers for the evolution of self-control, however, given the lack of data on many parrot species’ socio-ecological background, future research needs to employ a broader comparative approach to tackle these questions within the whole order of parrots (Psittaciformes).

## Supplementary Information

Below is the link to the electronic supplementary material.

Supplementary file1 (XLSX 27 kb)

Supplementary file2 (CSV 3 kb)

Supplementary file3 (CSV 177 kb)

Supplementary file4 (CSV 266 kb)

Supplementary file4 (MP4 79496 kb)

Supplementary file6 (TXT 4 kb)

## Data Availability

The raw data are available in the Supplementary Material.

## Code availability

The code is available in the Supplementary Material.
